# Roxadustat for the Treatment of Early Post-Transplantation Anemia

**DOI:** 10.3389/ti.2025.15033

**Published:** 2025-10-03

**Authors:** Louis Guenal, Philippe Gatault, Hélène Longuet, Lucie Maigret, Claire Ferran, Leïla Larbi, Alexandre Fillon, Jean-Michel Halimi, Matthias Büchler, Juliette Gueguen

**Affiliations:** ^1^ Service de Néphrologie-Hypertension Artérielle, Dialyses, Transplantation rénale, CHRU de Tours, Tours, France; ^2^ Faculté de Médecine de Tours, UMR 1327 ISCHEMIA, Tours, France

**Keywords:** kidney, transplantation, anemia, roxadustat, erythropoietin (EPO)

Dear Editors,

Early post-transplantation anemia (ePTA) is common in kidney transplant recipients (KTRs) and contributes to cardiovascular events, reduced quality of life, and overall mortality [1, 2]. Early PTA is driven by pre-transplant hemoglobin (Hb), preexisting deficiency (iron, folate, vitamin B12), intraoperative bleeding, inflammation, and delayed graft function [1]. Aside from blood transfusions, which should be avoided regarding the risk of developing HLA antibodies, the standard treatment for ePTA includes iron and vitamin supplementation, along with the use of recombinant erythropoietin (rEPO) [3, 4]. However, the efficacy of rEPO in ePTA remains uncertain, frequently observed in case of absolute or functional iron deficiency [1, 4, 5].

Roxadustat is an oral drug recently approved for treating anemia in chronic kidney disease (CKD). However, KTRs were excluded from clinical development studies [6, 7]. Roxadustat belongs to the class of hypoxia-inducible factor prolyl hydroxylase inhibitors (HIF-PHi). By modulating HIF, it exerts pleiotropic effects on the expression of genes involved in EPO synthesis, iron mobilization and inflammation, making it interesting for ePTA [8, 9]. We therefore implemented roxadustat as a routine treatment for ePTA instead of rEPO and report in this letter its efficacy and safety compared with rEPO used in the first month post transplantation.

We enrolled all consecutive patients receiving roxadustat for ePTA (defined as Hb <10g/dL during the first month after transplantation) and estimated glomerular filtration rate <60 mL/min/1.73 m^2^. Control group included all patients transplanted who received rEPO for ePTA during previous year. Data were retrieved from the ASTRE database, which prospectively collects data from KTRs (DR-2015-518). Additional data were adjudicated using medical records: Hb level, glomerular filtration rate, parathyroid hormone, blood transfusions, cardiovascular and thrombotic events, ferritin and transferrin saturation, C-reactive protein, date of treatment initiation, treatment dose and subsequent adjustments, and date of treatment discontinuation. Our protocol prioritizes correction of vitamin and iron deficiencies when indicated. Blood transfusions are performed in patients with Hb <7.5g/dL, or <9g/dL in the presence of symptoms or a history of vascular or cardiac stroke.

Roxadustat and rEPO were prescribed in accordance with the Summaries of Product Characteristics used for CKD. Roxadustat was started at 70 mg three times per week. Darbepoetin alfa was the single rEPO used, initiated at a dose of 0.45  μg/kg/week. In both groups, the dosage was increased in case of inefficacy at 1 month, the dosage was reduced or discontinued if Hb exceeded 12 g/dL or increased by more than 2 g/dL within 14 days. Efficacy was assessed by the proportion of patients achieving the target Hb level (>10 g/dL) and the need for blood transfusions beyond 2 weeks post-transplantation, to exclude the impact of perioperative bleeding. Safety was evaluated over the first 6 months, focusing on the incidence of severe adverse events, including vascular thrombosis, graft loss and major adverse cardiac events (4P-MACE).

Among 163 KTRs between May 2023 and July 2024, 46 received roxadustat for ePTA. Six patients were excluded (5 for early discontinuation of roxadustat unrelated treatment, 1 for concomitant use of rEPO). The prior year, 54 patients received rEPO for ePTA: 7 patients were excluded (5 for receiving fewer than 7 days of treatment, 1 for being under 18 years, 1 for bone marrow disease).

We observe that a greater proportion of patients in the roxadustat group achieved the target Hb of >10 g/dL at 3 months compared to the rEPO group (97% vs. 80% p = 0.04), which coincided with slightly higher Hb levels at that time point (Figure 1A). Moreover, a significantly higher number of patients treated with rEPO received transfusions beyond the first 2 weeks post-transplant (19.6% vs 2.6%, p = 0.02) (Figure 1B). At 6 months, 5 patients (15%) remained treated with roxadustat (28% in the rEPO group, p = 0.16), with a dose between 70 and 150 mg 3 times a week.

**FIGURE 1:**
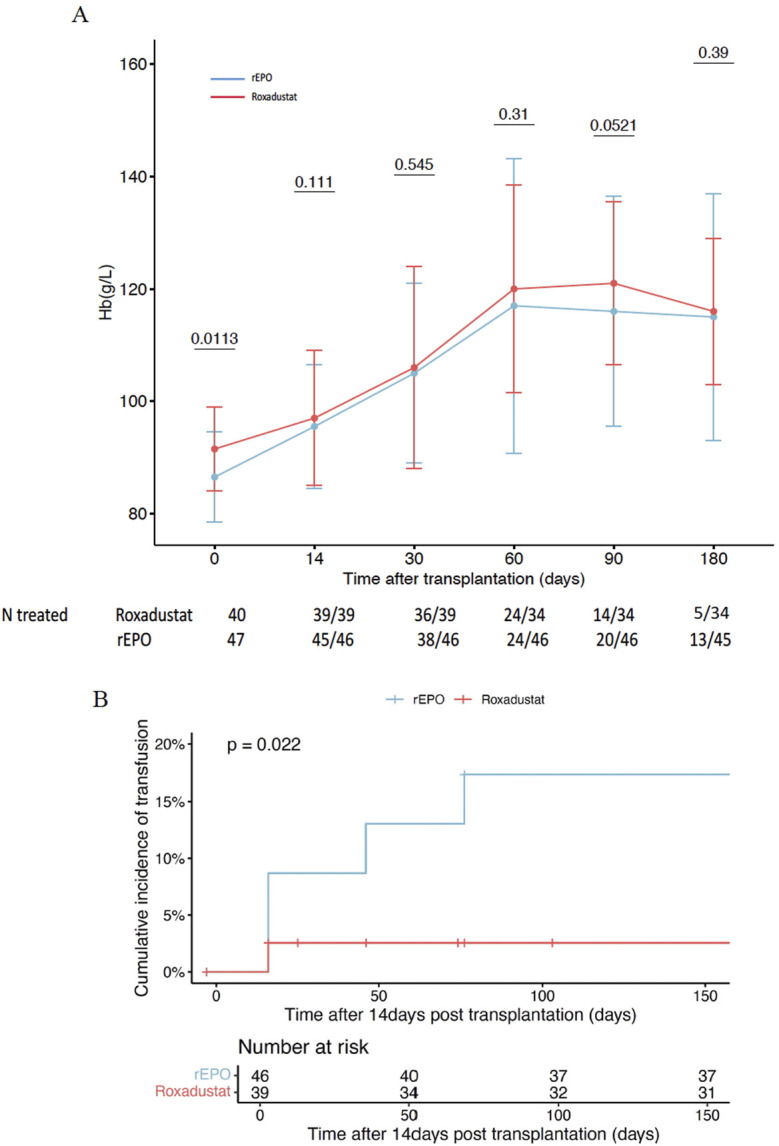
Efficacy of Roxadustat to treat early post transplant anemia in comparison to recombinant erythropoetin. **(A)** Hemoglobin level between roxadustat group and recombinant erythropoetin group. **(B)** Cumulative incidence of blood transfusion between roxadustat group and recombinant erythropoetin group. rEPO: recombinant erythropoetin, Hb: hemoglobin.

Globally, no difference was observed between both groups regarding safety outcomes. In the roxadustat group, 4 patients experienced thrombotic events (1 native kidney vein thrombosis, 2 lower limb deep vein thrombosis, and 1 arteriovenous fistula thrombosis); 2 patients experienced major cardiovascular events and 1 patient lost the graft due to severe artery stenosis leading to arterial thrombosis 6 months post-transplantation. In the rEPO group, 2 patients experienced thrombotic events (one lower limb deep vein thrombosis and one iliac vein thrombosis), no cardiovascular event nor graft loss was observed, 1 patient died from septic shock.

To our knowledge, we report the first European cohort study evaluating the efficacy and safety of roxadustat for the treatment of ePTA in KTRs. In contrast to randomized controlled trials conducted in patients with CKD which demonstrated non-inferiority of roxadustat compared to rEPO, our findings suggest that roxadustat may be more effective during the early post-transplantation period. At 3 months, 97% of patients in roxadustat group achieved the targeted Hb level, maintained at 6 months, with less need for transfusions compared to patients treated with rEPO. While the treatment was generally well tolerated, we call for particular caution regarding thrombotic risk, especially during the first 3 months following initiation. Although no statistically significant difference was observed, we suspect high level or a rapid increase of Hb to be a favoring factor of thrombosis. These adverse events are aligned with the European Medicines Agency’s recommendations for cautious use of roxadustat due to potential cardiovascular and thrombotic risks. These findings underscore the importance of closely monitoring Hb levels, especially at initiation and after each dose adjustment. Of note, 6/36 patients, who presented with persistently impaired graft function, were still receiving roxadustat at 6 months. This observation illustrates that some individuals may have a long-term indication for anemia treatment. It raises the question of long-term safety of roxadustat, particularly concerning its potential pro-angiogenic effects -through VEGF upregulation-in a population with an increased cancer risk. Nevertheless, randomized trials have not demonstrated an increased incidence of cancers, in line with findings from preclinical models [10]. This may be due to an incomplete activation of the HIF pathway, insufficient to trigger the VEGF gene expression and to the inhibition of tumor growth by modifying the microtumoral environment, as suggested by *in vitro* studies. In our opinion, this theorical risk should warrant vigilant monitoring rather than leading to the exclusion of KTRs.

In conclusion, our study suggests that the use of roxadustat may be more effective than rEPO in the management of ePTA in KTRs. However, its benefit-risk profile warrants further investigations in a randomized controlled trial.

## Data Availability

The original contributions presented in the study are included in the article/supplementary material, further inquiries can be directed to the corresponding author.

## Abbreviations

CKD, chronic kidney disease; ePTA, early post transplantation anemia; GFR, glomerular filtration rate; Hb, hemoglobin; HIF, hypoxia inducible factor; rEPO, recombinant erythropoietin; KDIGO, Kidney Disease Improval Global Outcomes; KTR, kidney transplant recipient; PHI, prolylhydroxylase inhibitor; rEPO: recombinant erythropoietin.
